# Incidence and predictive value of social frailty among community-dwelling older adults in Southwest China: A prospective cohort study

**DOI:** 10.3389/fpubh.2023.1103651

**Published:** 2023-02-20

**Authors:** Qian-qian Sun, Ke Tan, Hui-yu Tang, Yan-yan Liu, Huan Zhu, Hai Qin, Xin Xia, Min Zhang, Yan-yu Chen, Shuang-shuang Nie, Shuang Wang

**Affiliations:** ^1^The Center of Gerontology and Geriatrics, Sichuan University West China Hospital, Chengdu, Sichuan, China; ^2^National Clinical Research Center for Geriatrics, Sichuan University West China Hospital, Chengdu, Sichuan, China; ^3^Department of Epidemiology and Health Statistics, Sichuan University West China Fourth Hospital, Chengdu, Sichuan, China; ^4^Department of Clinic Development and Medical Affairs, Fosun Adgenvax Biopharmaceutical Co., Ltd., Chengdu, Sichuan, China; ^5^Internal Medicine Department, Pingyi Community Health Service Center, Dujiangyan, Sichuan, China; ^6^Department of Geriatrics, Sichuan Provincial People's Hospital, Sichuan Academy of Medical Science, Chengdu, Sichuan, China; ^7^Department of Rheumatology and Immunology, Chongqing Emergency Medical Center, Chongqing, China; ^8^Department of General Medicine, The Affiliated Qingdao Central Hospital of Qingdao University, Qingdao, Shandong, China

**Keywords:** social frailty (SF), community-dwelling older adults, adverse health events, mortality, prospective cohort study

## Abstract

**Background:**

Few studies have focused on the incidence and correlation of social frailty (SF) with adverse health events in Southwest China. This study aims to explore the predictive value of SF for adverse health events.

**Methods:**

A 6-year prospective cohort study was employed, a total of 460 community-dwelling older adults aged 65 years and above were analyzed to provide a baseline in 2014. Participants completed two longitudinal follow-ups at 3 (2017, 426 participants involved) and 6 (2020, 359 participants involved) years later. A modified social frailty screening index was used in this study, and adverse health events such as physical frailty (PF) deterioration, disability, hospitalization, falls, and mortality were evaluated.

**Results:**

Among these participants in 2014, the median age was 71 years, 41.1% were male, and 71.1% were married or cohabiting, up to 112 (24.3%) of them were classified as SF. It was observed that aging (OR = 1.04, 95% CI = 1.00–1.07, *P* = 0.047) and having family members die in the past year (OR = 2.60, 95% CI = 0.93–7.25, *P* = 0.068) were risk factors of SF, whereas having a mate (OR = 0.40, 95% CI = 0.25–0.66, *P* = 0.000) and having family members to help with care (OR = 0.53, 95% CI = 0.26–1.11, *P* = 0.092) were protective factors of SF. The cross-sectional study demonstrated that SF was only significantly associated with disability (OR = 12.89, 95% CI = 2.67–62.13, *P* = 0.001) at wave 1. Baseline SF significantly explained the incidence of mortality at the 3-year (medium-term, OR = 4.89, 95% CI = 2.23–10.71, *P* = 0.000) and 6-year follow-ups (long-term, OR = 2.22, 95% CI = 1.15–4.28, *P* = 0.017).

**Conclusion:**

SF prevalence was higher in the Chinese older population. Older adults with SF had a significantly increased incidence of mortality at the longitudinal follow-up. Consecutive comprehensive health management of SF (e.g., avoiding living alone and increasing social engagement) is urgently needed for the purposes of early prevention and multidimensional intervention in adverse health events, including disability and mortality.

## Introduction

Among the worldwide aged population, frailty is an important health issue and is characterized by decreased physiological reserve and function across multiple physiologic systems (1, 2). It is associated with adverse events, including falls (3, 4), hospitalization, institutionalization (5), disability (6), lower quality of life and mortality (7). As a part of abnormal aging, frailty is a common public health problem with a prevalence of about 10% in the community-dwelling elderly population (1). Frailty has several phenotypes, such as physical, cognitive, psychological, nutritional and social frailty (8). Compared with other frail phenotypes, social frailty (SF) is the most unexplored component (9) because of the inconsistency of definition and measurement way of SF (10). Even though, the prevalence of SF were reported ranged from 7.7% (China), 11.1 or 18.0% (Japan) to 18.4% (Singapore) (9, 11–13) based on different screening tools. Therefore, social frailty is also accepted as an abnormal process of aging which contributed to disability (14), cognition impairment, depression (11), and mortality (15, 16), as same as physical frailty.

As for the screening tools, seven-item SF index was first constructed by Teo et al. (13) based on the Singapore Longitudinal Aging Studies Wave 1 (SLAS-1) cohort. However, this assessment method was time-consuming in practice. Bessa et al. (17) attempted to give an integrated conceptualization of SF which covered four aspects: measures general, social resources, social behaviors, and the satisfaction of basic social requirements (18). Then a modified SF index screen tool (19) based on those conception was developed by Nagai et al. (20) in Japan, who confirmed that briefly SF can predict future incidents of activity limitation and mortality in community-dwelling older adults (15). Yet, although the cultural may vary, the understanding of SF and its mechanisms remains the same; although general and social resources as well as social behaviors or activities may vary among different countries and cultures, they are still contribute to the social needs fulfillment (21). Considering that older adults must increasingly rely on their social relationships and social environment due to policy measures aimed at reducing the financing of formal care and support, the incidence of SF and its effect on adverse health events becomes even more important (21).

China has the largest older population in the world (8). China is a country that is changing rapidly including family cohesion and traditional family-based social support considerably weakened, which might contribute to the score of the SF index (16). However, the SF of older individuals in Chinese communities varies greatly, and few studies have reported the correlation of SF with adverse health events in Southwest China. Therefore, the core aim of this study was to identify the incidence of SF by using a modified SF index assessment tool and to explore the relationship between SF and deterioration of PF, disability, hospitalization, falls and all-cause mortality among community-dwelling older adults in both cross-sectional and longitudinal studies.

## Methods

### Study design, setting, and participants

**Figure 1:**
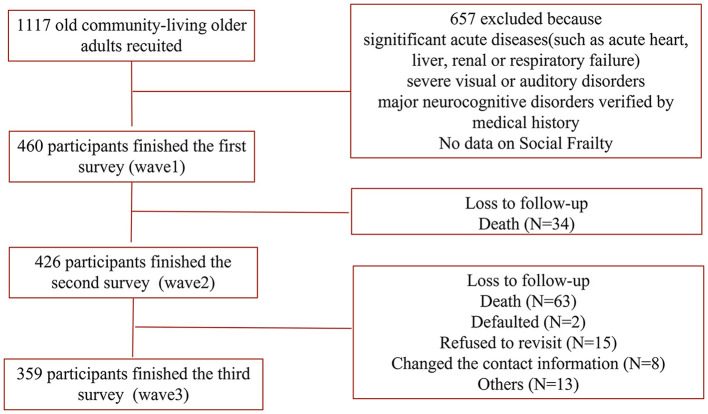
Flow chart demonstrating the process used to select the study participants.

All data were obtained from “the Survey on the Disease, Psychology and Social Support of the older Community-dwelling Population in Dujiangyan,” a prospective cohort study for older individuals aged 65 years and older in China supported by Johnson & Johnson global novel project (2013), which has been described in detail in our previous study (22). The exclusion criteria included: (1) any disease in the acute phase cause life expectancy <6 months, such as acute heart, liver, kidney and respiratory failure were excluded for better follow-up; (2) severe visual/hearing impairment and severe dementia; and (3) unwillingness to be investigated and unable to communicate with the investigators. In Figure 1, a total of 1,117 older adults were first recruited in January 2014. Among them, 460 older adults finished the first survey (wave 1). The next two waves of follow-up were conducted in January 2017 (426 participants involved, wave 2) and January 2020 (359 participants involved, wave 3). The research protocol was reviewed and approved by the Ethics Review Committee of Sichuan University (registration number 2014-206), and informed written consent was obtained from all participants.

### Measurements

#### Definition of social frailty

Taking the practicable and available into consideration, a modified social frailty index (15) (general and social resources, social behaviors, and the satisfaction of basic social requirements) was used to assess social frailty in this study, which was described as follows: (1) financial support: “Is your annual per capita income of households <RMB10,000?” (yes = 1 point, no = 0 points), (2) living status: “How many people do you live with?” (0 = 1 point, ≥1 = 0 points), (3) social activity: “Do you participate in any community activities regularly?” (no = 1 point, yes = 0 points), and (4) social contact: “Do you sometimes visit your friends and relatives?” (no = 1 point, yes = 0 points). Participants with summed scores of 2 or more were categorized as having SF. A score of 0–1 was regarded as non-social frailty.

#### Assessment of physical frailty

Physical frailty was defined using the Frail Scale comprising five components (18): fatigue, resistance, ambulation, illness, and loss of weight. Components were operationalized. Participants were considered physically frail (score = 3–5), prefrail (score = 1–2), and robust (score = 0) according to the summed score.

#### Adverse health events

Deterioration of PF was defined as from robust to pre-frail or frail and from pre-frail to frail.

Disability was defined as requiring assistance on one or more IADL (instrumental activities of daily living, eight items) or ADL (activities of daily living, seven items) item(s).

Hospitalization was assessed *via* self-reported hospitalizations or reviewing computerized HIS records from 2014 to 2020.

Falls were defined as any sudden drop from one surface to a lower surface. We assessed falls by asking the participants: “Did you fall in the previous year?” (response categories “yes” and “no”).

Mortality data were collected by local government records or family members' self-reports.

#### Other covariates

A personal information questionnaire was used to collect the participants' characteristics. The sociodemographic covariates of participants included age, gender, marital status (with or without life partner), education level (according to the International Standard Classification of Education), self-rated sleep quality, self-reported memory status (good, average, and poor), Residency Period (<3, 3–10, >10 years), family member has died in 1 year. Social characteristics were assessed through the following dimensions: medical service support (medical insurance), expenditure (in debt), social engagement (whether having family members to help with care), and emotional support (willingness to make friends and with a confidant in one's life). Geriatric syndrome (malnutrition, depression, cognitive impairment, comorbidity, and polypharmacy) and physical profile (HbA1c, BMI, systolic BP, and diastolic BP).

### Statistical analysis

The Statistical Package for Social Science (SPSS) for Windows version 21.0 (SPSS, Chicago, IL, USA) was used to calculate descriptive statistics and to obtain the frequency and percentage distributions. The characteristics of participants' at baseline were compared by using Mann–Whitney *U*, or chi-square tests according to the type of variables. Multivariate logistic regression using forward stepwise regression (*P* < 0.10 for variable inclusion criteria) were conducted with the aim of examining the association of SF with adverse health events in cross-sectional and predictive value of SF on adverse events in longitudinal scenarios.

## Results

### Participant characteristics

Table 1 summarizes the overall SF status and sociodemographic characteristics of participants at wave 1. Among these 460 participants in 2014, the median age was 71 years, 41.1% were male, and 71.1% were married or cohabiting. During the wave 2 follow-up, 34 participants died. The wave 3 visit and assessment were conducted in January 2020, during which 359 participants were completed, and 63 died. Another 38 participants were excluded as they defaulted (*n* = 2), refused to revisit (*n* = 15), changed the contact information (*n* = 8), and other (*n* = 13; Figure 1).

**Table 1:** Demographic characteristics of SF in wave 1 (*N* = 460).

**Variables**	**N-SF (*N* = 348)**	**SF (*N* = 112)**	**Total**	***P***
Age (years)	71 (67–76)	73 (69–79.75)	71.0 (67.0, 77.0)	0.303
Sex (males; %)	200 (57.5)	71 (63.4)	189 (41.1)	0.268
Marital status (having a mate; %)	270 (77.6)	57 (50.9)	327 (71.1)	**0.000**
Education				**0.001**
Illiterate (%)	61 (17.5)	38 (33.9)	99 (21.5)	
Elementary school (%)	143 (41.1)	41 (36.6)	184 (40.0)	
Middle school or higher (%)	144 (41.4)	33 (29.5)	177 (38.5)	
Sleep quality (bad; %)	177 (50.9)	63 (56.3)	240 (52.2)	0.321
Self-reported memory status				0.944
Good (%)	92 (26.9)	30 (27.5)	131 (28.5)	
Normal (%)	138 (40.4)	42 (38.5)	180 (39.1)	
Bad (%)	112 (32.7)	37 (33.9)	149 (32.4)	
Residency period (year; %)				0.553
≤ 3	118 (34.0)	42 (37.5)	161 (35.0)	
3 <x ≤ 10	101 (29.1)	35 (31.3)	136 (29.6)	
>10	128 (36.9)	35 (31.3)	163 (35.4)	
Family member has died in 1 year (%)	10 (2.9)	8 (7.1)	18 (3.9)	**0.043**
With a confidant (%)	326 (93.9)	99 (88.4)	425 (92.4)	**0.051**
Having family members to take care (%)	63 (18.1)	11 (9.8)	74 (16.1)	**0.038**
SF four components				
Living alone (%)	19 (5.5)	38 (33.9)	57 (12.5)	**0.000**
Lack of social activity (%)	24 (6.9)	68 (60.7)	92 (20)	**0.000**
Lack of contact with neighbors (%)	147 (42.2)	102 (91.1)	249 (54.1)	**0.000**
Financial difficulties (%)	20 (5.8)	44 (39.3)	64 (14.0)	**0.000**
Physical frailty (PF)				0.173
Robust (%)	182 (52.3)	52 (46.4)	234 (50.9)	
Pre-frail (%)	148 (42.5)	49 (43.8)	197 (42.8)	
Frail (%)	18 (5.2)	11 (9.8)	29 (6.3)	
Adverse health events				
Disability (%)	3 (0.9)	11 (9.8)	14 (3.0)	**0.000**
Hospitalization within past 1 year (%)	170 (48.9)	63 (56.3)	74 (16.1)	0.173
Fall	25 (7.2)	8 (7.1)	33 (7.2)	0.988
Geriatric syndrome				
Malnutrition (%)	36 (10.3)	17 (15.2)	53 (11.5)	0.163
Depression (%)	9 (2.6)	5 (4.5)	14 (3.0)	0.490
Cognitive impairment (%)	39 (11.2)	25 (22.3)	64 (13.9)	**0.003**
Comorbidity	138 (39.7)	40 (35.7)	178 (38.7)	0.456
Polypharmacy	33 (9.5)	7 (6.3)	40 (8.7)	0.291
Physical profile				
HbA1C (%)	5.7 ± 0.8	5.7 ± 0.9	5.7 ± 0.8	0.540
BMI (kg/m^2^)	24.8 ± 3.5	24.2 ± 3.5	24.6 ± 3.5	0.107
Systolic BP (mmHg)	139.5 ± 18.7	139.5 ± 19.5	139.5 ± 18.9	0.987
Diastolic BP (mmHg)	80.4 ± 11.0	81.9 ± 10.9	80.8 ± 11.0	0.188

SF, Social Frailty; PF, Physical Frailty; BMI, body mass index; HbA1c, glycated hemoglobin; BP, Blood Presure.

p-values < 0.05 are printed in bold.

### Prevalence and risk factors for SF

The prevalence of SF increased with time and was 24.3% (112/460, wave 1), 28.9% (123/426 wave 2), and 60.4% (217/359 wave 3). During the two waves of follow-up, we observed a significant increase in SF deterioration [from 72 (15.7%) to 154 (42.9%)], and only 23 (6.4%) participants had improved in wave 3. A high prevalence of SF was observed among participants who were older, without a mate, had lower levels of education, with family members died in the last year, had family members to take care, who lacked social activity, who lacked contact with neighbors, had financial difficulties, had a disability or cognitive impairment. There were no significant difference between the two groups in terms of who had the worse sleep patterns, physical frailty, number of falls, hospitalizations within the past year and other geriatric syndromes (Tables 1, 2).

**Table 2:** The overall social frailty status and adverse health events of participants at each visit.

**Variables**	**2014 (wave 1; *N* = 460)**	**2017 (wave 2; *N* = 426)**	**2020 (wave 3; *N* = 359)**
**Social frailty (%)**	112 (24.3)	123 (28.9)	217 (60.4)
Four components			
Living alone (%)	57 (12.5)	89 (20.9)	89 (24.8)
Lack of social activity (%)	92 (20)	147 (34.5)	266 (74.1)
Lack of contact with	249 (54.1)	126 (29.6)	201 (56.0)
neighbors (%)			
Financial difficulties (%)	64 (14.0)	72 (16.9)	36 (10.0)
**Adverse health events**			
Transitions in SF			
Deterioration (%)	-	72 (15.7)	154 (42.9)
Unchanging (%)	-	327 (71.1)	182 (50.7)
Improve (%)	-	61 (13.3)	23 (6.4)
Transitions in PF			
Deterioration (%)	-	67 (15.7)	78 (21.7)
Unchanging (%)	-	212 (49.8)	195 (54.3)
Improve (%)	-	147 (34.5)	86 (24.0)
Disability (%)	14 (3.0)	40 (9.4)	81 (22.6)
Hospitalization within past 1 year (%)	233 (50.7)	207 (48.6)	158 (44.0)
Fall (%)	33 (7.2)	29 (6.8)	96 (26.7)
Mortality (%)	-	34 (7.4)	63 (13.7)

SF, Social Frailty; PF, Physical Frailty.

**Figure 2:**
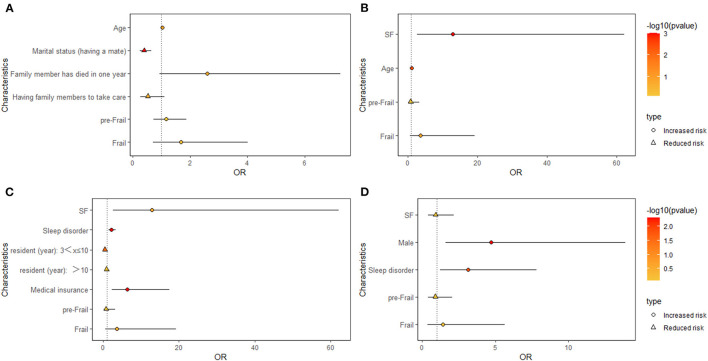
Forest plots showing the analysis of risk factors associated with SF **(A)** and cross-sectional analyses of baseline SF with adverse health events in wave 1 **(B–D)**. **(B)** Cross-sectional analyses of baseline SF with disability. **(C)** Cross-sectional analyses of baseline SF with hospitalization. **(D)** Cross-sectional analyses of baseline SF with fall.

Multivariate logistic regression analysis was used to determine the possible associated factors for SF (wave 1) in Figure 2A. The risk factors of SF were significantly associated with aging (OR = 1.04, 95% CI = 1.00–1.07, *P* = 0.047) and having family members die in the past year (OR = 2.60, 95% CI = 0.93–7.25, *P* = 0.068), whereas, having a mate (OR = 0.40, 95% CI = 0.25–0.66, *P* = 0.000) and having family members to help with care (OR = 0.53, 95% CI = 0.26–1.11, *P* = 0.092) were protective factors of SF.

### The incidence of different adverse health events

The prevalence of adverse health events from wave 1 to wave 3 (Table 2) was described as follows: disability was 3.0, 9.4, and 22.6%; falls were 7.2, 6.8, and 26.7%; hospitalization within the past 1 year was 50.7, 48.6, and 44.0%; and mortality was 7.4% (wave 2) and 13.7% (wave 3). In wave 2, the number of disabilities [40 (9.4%)] markedly increased, while the rates of falls [29 (6.8%)] and hospitalization [207 (48.6%)] were comparable to those in wave 1. By the end of wave 3, it was observed that 78 (21.7%) of patients had worsened into PF or pre-PF. A significant increase in the proportion of disabilities (22.6%), falls (26.7%) and mortality (13.7%) after 6 years were observed, but hospitalization (44.0%) was further decreased.

### Relationship of SF with adverse health events: Cross-sectional and longitudinal analysis

Figures 2B–D presents the cross-sectional association of SF with adverse health events. At the first wave visit, after adjusting for background characteristics and adverse events, the logistic regression analyses demonstrated that SF was significantly associated with disability (OR = 12.83, 95% CI = 2.67–62.13, *P* = 0.001) and age (OR = 1.12, 1.04–1.21, *P* = 0.004). Poor sleep quality (OR = 2.20, 95% CI = 1.43–3.36, *P* = 0.000), residency period (3<x≤10 years; OR = 0.48, 95% CI = 0.29–0.78, *P* = 0.003) and medical insurance (OR = 6.41, 95% CI = 2.34–17.57, *P* = 0.000) were related to hospitalization. Male participants (OR = 4.73, 95% CI = 1.62–13.87, *P* = 0.005) and those with poor sleep quality (OR = 3.12, 95% CI = 1.24–7.83, *P* = 0.015) at baseline had an increased risk of falling. However, baseline PF was not significantly associated with an increased risk of disability, hospitalizations or fall incidents.

Longitudinal analyses (waves 2) showed that SF significantly predicted the incidence of mortality (OR = 4.89, 95% CI = 2.23–10.71, *P* = 0.000), whereas SF did not have a significant effect on disability, hospitalizations, falls or PF deterioration. At the third wave of follow-up, the multivariate logistic regression analysis indicated that baseline SF significantly increased the risk of 6-year mortality (OR = 2.22, 95% CI = 1.15–4.28, *P* = 0.017). However, baseline SF was significantly associated with a decreased risk of hospitalization (OR = 0.57, 95% CI = 0.33–0.98, *P* = 0.041). No significant correlations with disability, falls or PF deterioration were found (Table 3, Supplementary Tables 1, 2).

**Table 3:** Longitudinal analyses of SF with adverse health events.

**Variables**	**Follow-up**		**Longitudinal analysis (wave 2)**	**Longitudinal analysis (wave 3)**
PF deterioration	Sig.		0.442	0.903
	Exp (B)		0.75	1.04
	95%	Lower	0.36	0.54
	C.I.	Upper	1.56	2.01
Disability	Sig.		0.142	0.285
	Exp (B)		1.95	0.71
	95%	Lower	0.8	0.37
	C.I.	Upper	4.73	1.34
Hospitalization	Sig.		0.85	**0.041**
	Exp (B)		1.05	0.57
	95%	Lower	0.63	0.33
	C.I.	Upper	1.75	0.98
Fall	Sig.		0.612	0.303
	Exp (B)		0.78	0.74
	95%	Lower	0.3	0.41
	C.I.	Upper	2.04	1.32
Mortality	Sig.		**0.000**	**0.017**
	Exp (B)		4.89	2.22
	95%	Lower	2.23	1.15
	C.I.	Upper	10.71	4.28

SF, Social Frailty; PF, Physical Frailty.

We used the stepwise logistic regression model to analyze the risk factors of SF. p-values < 0.05 are printed in bold.

## Discussion

SF in aging populations is of grave concern because of social issues faced by older individuals, such as those related to income, family dynamics, and social inclusion (15). As a complex concept, SF is comprehensive, and there is still no consensus on the criteria. Comparing with some social terms, such as social isolation and social support alone, more directions (21) were taken into account. Among them, living alone and infrequent contact with family or friends might be the core of social frailty. So, the modified SF screen tool that consist of general and social resources (“is your annual per capita income of households <RMB10,000?”), social behaviors (“Do you participate in any community activities regularly?” and “Do you sometimes visit your friends and relatives?”), and the satisfaction of basic social requirements (“How many people do you live with?”) was (21) chose in this study.

Our study found that the prevalence of SF in older individuals was 24.3%. At the end of our study, 60.4% of older adult participants were found to have SF. the baseline prevalence was higher than that of Ma et al. (16), who found that the prevalence of SF was 7.7% (aged ≥ 60 years) in Beijing by HALFT scale (inability to help others, limited social participation, loneliness, financial difficultly, and not having anyone to talk to) in 2004, 11.1% (mean age 71.9 years) in Japan (11) by the 5-item scale (living alone, going out less frequently compared to last year, visiting friends sometimes, feeling helpful to friends or family, and talking with someone every day) or 18.0% (mean age 75.2 years) by modified SF index screening questionnaire (financial support, living status, social activity, and social contact) (15), and 18.4% (mean age 66.1 years) in Singapore (13) by the Seven-item social frailty index (living alone, no education, absence of a confident, infrequent contact, infrequent social activities, financial difficulty and socioeconomic deprivation). Furthermore, our study reported the status changes of SF over time: half of participants had SF status stable (50.7%) while half deterioration (42.9%) at the end of 6 years. The potential reasons of high prevalence of SF in this study might be: (1) the mean age of our participants were older (mean age 71 years) than other studies; (2) reduced social activity and social contact by unique earthquake in 2008. Some older adults have to move to the present place of residence during the reconstruction of the disaster. They had fewer relatives and friends than before; (3) with the deterioration of aging and economic development of society, traditional family-based social support given to older community-dwelling adults was weakened gradually, the left-behind co-habitants were spouse or older adults were lived alone which contributed the living status changing; and (4) most of our participants were older adults lived in Urban-rural fringe, financial support was relatively limited (11).

We analysis the risk factor for SF by using baseline data. The results showed that participants with advanced age, marital status of no partner, lower education and cognitive impairment had a high prevalence of SF. Older age is a risk factor of SF An obviously increased proportion of SF was found in age group between 80 and 84 years old, which amounted to 22.0% and was even higher than 41.8% in patients 85 years of age and older (11). From this perspective, older age itself seems to be a risk factor of SF to come into being. Participants with a marital status of no partner were prone to isolation and loneliness, both linked to SF. It has been suggested that a decline in cognitive function may occur concurrently with the presence of SF (23), which may lead to adverse health events, such as mortality, hospitalization, falls and disability. In addition, the study also found that aging (OR = 1.04, 95% CI = 1.00–1.07, *P* = 0.047) and having a family member who died within 1 year (OR = 2.60, 95% CI = 0.93–7.25, *P* = 0.068) were negative factors of SF, while having a mate (OR = 0.40, 95% CI = 0.25–0.66, *P* = 0.000) and having family members to help with care (OR = 0.53, 95% CI = 0.26–1.11, *P* = 0.092) were protective factors for SF in the Chinese culture. In the clinical setting, understanding who is more likely to deteriorate and who may remain stable, or even revert back to the better state, will allow clinicians to focus on those at the highest risk for early interventions (24). Despite many studies determining the effects of interventions on PF, the number of studies on interventions that target SF is limited (25). This study found that avoiding living alone (having a partner) and increasing social engagement (having family members to help with care) can contribute to preventing SF.

Furthermore, this study also provides evidence on the relationship between SF and adverse health events in both the medium- and long-term future. First, based on this cross-sectional analyses, it was found that SF was significantly correlated with disability and hospitalization. No relationship was found between falls and PF after adjusting for all the other variables in the model. In some studies, they found that the number of disabled persons among SF older increased by 2.30 times, and the number of severely disabled persons increased by 6.27 times (13). Other studies also found that SF status is negatively associated with physical functioning (26) and is associated with a higher incidence of disability (27). This study verified that SF, as an indicator of a decline in social function (28), is a risk factor for dependency (29). It shows that participants with baseline SF are ~12 times more likely to have an incident related to disability than participants who are not SF. Some factors, such as age, male sex, poor sleep quality, medium period residence, and medical insurance, were also associated with adverse health outcomes, and these results were consistent with other studies (30–33).

Second, the regression analyses (longitudinal) revealed that SF was significantly associated with mortality during wave 2 (medium-term, OR = 4.89, 95% CI = 2.23–10.71, *P* = 0.000) and wave 3 (long-term, OR = 2.22, 95% CI = 1.15–4.28, *P* = 0.017), and medium-term mortality was higher than long-term mortality. Ma et al. (16) examined the correlation between SF and mortality among community-dwelling older adults. After adjusting for age and sex, the 8-year mortality hazard ratios were 2.5–4.3 for those with SF, and SF predicted 8-year mortality. Yamada et al. (15) conducted a prospective cohort study in 6,603 community-dwelling adults aged 65 years and older who were living independently in a city in Shiga prefecture in 2011 and found that 48.5% of those with SF died, indicating that community-dwelling older adults with SF (adjusted HR 1.71, 95% CI 1.54–1.90) were at higher risk of death over 6 years. Our data were higher than those studies, and participants with SF had a 4- and 2-fold incidence of mortality than those without SF, which was consistent with those results. Mortality is the most important variable among adverse health outcomes.

Third, some studies found that social factors could be associated with an increased incidence of hospitalizations. Social factors of self-neglect have been linked to poor social support, reduced nutritional intake and physical function (34), resulting in poor quality of life and increased falls, hospitalization and mortality. In a sample of 963 Brazilian people (35) aged 60 years and older, the TFI predicted hospitalization. However, in this study, baseline SF was significantly associated with a decreased risk of hospitalization (OR 0.57, 95% CI = 0.33–0.98, *P* = 0.041) during the 6-year follow-up. It is speculated that the baseline SF individuals were prone to having lower incomes and did not have equal hospitalizations; therefore, SF individuals in Southwest China predicted a decreased risk of hospitalization longitudinally.

Impaired falls in the older are a major source of injury resulting in disability (36). Although multiple longitudinal studies have investigated frailty as a predictor of future falls, the results were mixed (37). Gobbens et al. (38) found that SF was only correlated with disability and falls in a sample of 180 Dutch community-dwelling older people aged 70 years and older. The future fall risk according to frailty seemed to be higher in men than in women. SF is a factor associated with accelerated decline in both physical and mental functioning. In addition, it has been suggested that social roles gradually decrease before a decline in cognitive and physical functioning is reported (25). Makizako et al. (9) found that participants who were SF at baseline had an increased risk of developing PF (OR = 3.93, 95% CI = 1.02–15.15) and physical prefrailty (OR = 2.50, 95% CI = 1.30–4.80). This indicates that those who are SF may be at greater risk of developing PF in the near future. However, this longitudinal study did not find that SF predicts PF deterioration in mid- and long-term studies. The reason for the lack of a relationship with falls and PF deterioration may be that there were more women among participants at the baseline. Another reason may be that participants came from the urban-rural fringe areas; many were labor workers and had better physical fitness.

In addition to SF, the current study also found that those resident <3 years and without a confidant also had an increased risk of developing adverse health events, such as falls and mortality. In the future, these factors should be taken into account as supplementary components of SF screening tools. This modified tool may better detect adverse health events, but it may need further validation.

This study also has some limitations. First, the instrument used to evaluate SF was self-reported, so it may be subject to potential recall bias despite all the questionnaires were conducted face-to-face one by one at all waves, and all investigators participated in the study were trained by standard protocol, so that the subjects had no understanding error. Second, due to the vary widely across regions and smaller geographic and cultural units, perhaps we miss an opportunity to take full advantage of this framing to educate the world outside China about those changes. Finally, considering these limitations, further studies will be needed to explore a consecutive comprehensive health management of SF for the purposes of early prevention and multidimensional intervention in adverse health outcomes.

## Conclusion

This study reported the incidence of SF and its associated factors and highlights the predictive values of SF on adverse health events longitudinally. First, the incidence of SF was higher and its transitions was the majority of participants remained SF status stable or deteriorated at the end of 6 years in community-dwelling older adults in Southwest China. Second, this study found that avoiding living alone (having a partner) and increasing social engagement (having family members to help with care) can contribute to SF. Finally, older adults with SF had a significantly increased incidence of mortality at the longitudinal follow-up. Consecutive comprehensive health management of SF (e.g., avoiding living alone, increasing social engagement) is urgently needed for the purposes of early prevention and multidimensional intervention in adverse health events. The present study provided new, additional evidence for assessing SF in Chinese community-dwelling older people aiming to prevent or delay adverse events, including disability, hospitalization, and mortality.

## Data availability statement

The raw data supporting the conclusions of this article will be made available by the authors, without undue reservation.

## Ethics statement

The studies involving human participants were reviewed and approved by the Ethics Review Committee of Sichuan University. The patients/participants provided their written informed consent to participate in this study.

## Author contributions

Q-qS wrote the manuscript and participated in all aspects of this research. KT, H-yT, Y-yL, HZ, and XX assisted with study design, data analysis, and interpretation. HQ, MZ, Y-yC, and S-sN helped in collecting data. SW reviewed the final article. All authors: revision of manuscript for important intellectual content and approval of final draft.

## References

[1] Collard RM Boter H Schoevers RA Oude Voshaar RC. Prevalence of frailty in community-dwelling older persons: A systematic review. J Am Geriatr Soc. (2012) 60:1487–92. 10.1111/j.1532-5415.2012.04054.x 22881367

[2] Clegg A Young J Iliffe S Rikkert MO Rockwood K. Frailty in elderly people. Lancet. (2013) 381:752–62. 10.1016/S0140-6736(12)62167-9 23395245 PMC4098658

[3] Cheng MH Chang SF. Frailty as a risk factor for falls among community dwelling people: Evidence from a meta-analysis. J Nurs Scholarsh. (2017) 49:529–36. 10.1111/jnu.12322 28755453

[4] Del BruttoOHMeraRMPeinadoCDZambranoMSedlerMJ. Frailty and risk of falls in community-dwelling older adults living in a rural setting. The Atahualpa project. J Frailty Aging. (2020) 9:150–4. 10.14283/jfa.2019.3632588029

[5] Kojima G. Frailty as a predictor of nursing home placement among community-dwelling older adults: A systematic review and meta-analysis. J Geriatr Phys Ther. (2018) 41:42–8. 10.1519/JPT.0000000000000097 27341327

[6] Kojima G. Frailty as a predictor of disabilities among community-dwelling older people: A systematic review and meta-analysis. Disabil Rehabil. (2017) 39:1897–908. 10.1080/09638288.2016.1212282 27558741

[7] Kojima G Iliffe S Walters K. Frailty index as a predictor of mortality: A systematic review and meta-analysis. Age Ageing. (2018) 47:193–200. 10.1093/ageing/afx162 29040347

[8] Rockwood K. What would make a definition of frailty successful? Age Ageing. (2005) 34:432–4. 10.1093/ageing/afi146 16107450

[9] Makizako H Shimada H Doi T Tsutsumimoto K Hotta R Nakakubo S. Social frailty leads to the development of physical frailty among physically non-frail adults: A four-year follow-up longitudinal cohort study. Int J Environ Res Public Health. (2018) 15:30490. 10.3390/ijerph15030490 29534470 PMC5877035

[10] Quach LT Primack J Bozzay M Madrigal C Erqou S Rudolph JL. The intersection of physical and social frailty in older adults. R I Med J. (2021) 104:16–9. 33926153 PMC10274204

[11] Tsutsumimoto K Doi T Makizako H Hotta R Nakakubo S Makino K. Association of social frailty with both cognitive and physical deficits among older people. J Am Med Dir Assoc. (2017) 18:603–7. 10.1016/j.jamda.2017.02.004 28411094

[12] Makizako H Shimada H Tsutsumimoto K Lee S Doi T Nakakubo S. Social frailty in community-dwelling older adults as a risk factor for disability. J Am Med Dir Assoc. (2015) 16:1003 e7–11. 10.1016/j.jamda.2015.08.023 26482055

[13] Teo N Gao Q Nyunt MSZ Wee SL Ng TP. Social frailty and functional disability: Findings from the Singapore longitudinal ageing studies. J Am Med Dir Assoc. (2017) 18:637 e13–637 e19. 10.1016/j.jamda.2017.04.015 28648903

[14] Teo N Yeo PS Gao Q Nyunt MSZ Foo JJ Wee SL. A bio-psycho-social approach for frailty amongst Singaporean Chinese community-dwelling older adults—Evidence from the Singapore Longitudinal Aging Study. BMC Geriatr. (2019) 19:350. 10.1186/s12877-019-1367-9 31830924 PMC6909571

[15] Yamada M Arai H. Social frailty predicts incident disability and mortality among community-dwelling Japanese older adults. J Am Med Dir Assoc. (2018) 19:1099–103. 10.1016/j.jamda.2018.09.013 30471801

[16] MaLSunFTangZ. Social frailty is associated with physical functioning, cognition, and depression, and predicts mortality. J Nutr Health Aging. (2018) 22:989–95. 10.1007/s12603-018-1054-030272104

[17] Bessa B Coelho T Ribeiro O. Social frailty dimensions and frailty models over time. Arch Gerontol Geriatr. (2021) 97:104515. 10.1016/j.archger.2021.104515 34597877

[18] Morley JE Malmstrom TK Miller DK. A simple frailty questionnaire (FRAIL) predicts outcomes in middle aged African Americans. J Nutr Health Aging. (2012) 16:601–8. 10.1007/s12603-012-0084-2 22836700 PMC4515112

[19] Armstrong JJ Andrew MK Mitnitski A Launer LJ White LR Rockwood K. Social vulnerability and survival across levels of frailty in the Honolulu-Asia Aging Study. Age Ageing. (2015) 44:709–12. 10.1093/ageing/afv016 25758407 PMC4476846

[20] Nagai K Tamaki K Kusunoki H Wada Y Tsuji S Itoh M. Physical frailty predicts the development of social frailty: A prospective cohort study. BMC Geriatr. (2020) 20:403. 10.1186/s12877-020-01814-2 33054731 PMC7557012

[21] Bunt S Steverink N Olthof J van der Schans CP Hobbelen MJS. Social frailty in older adults: A scoping review. Eur J Ageing. (2017) 14:323–34. 10.1007/s10433-017-0414-7 28936141 PMC5587459

[22] Tang H Zhu H Sun Q Qin H Wang S. Transitions in the cognitive frailty states in community-living older adults: A 6-year prospective cohort study. Front Aging Neurosci. (2021) 13:774268. 10.3389/fnagi.2021.774268 34924997 PMC8672135

[23] Makizako H Tsutsumimoto K Shimada H Arai H. Social frailty among community-dwelling older adults: Recommended assessments and implications. Ann Geriatr Med Res. (2018) 22:3–8. 10.4235/agmr.2018.22.1.3 32743237 PMC7387641

[24] Lee JS Auyeung TW Leung J Kwok T Woo J. Transitions in frailty states among community-living older adults and their associated factors. J Am Med Dir Assoc. (2014) 15:281–6. 10.1016/j.jamda.2013.12.002 24534517

[25] Dedeyne L Deschodt M Verschueren S Tournoy J Gielen E. Effects of multi-domain interventions in (pre)frail elderly on frailty, functional, and cognitive status: A systematic review. Clin Interv Aging. (2017) 12:873–96. 10.2147/CIA.S130794 28579766 PMC5448695

[26] Pek K Chew J Lim JP Yew S Tan CN Yeo A. Social frailty is independently associated with mood, nutrition, physical performance, and physical activity: Insights from a theory-guided approach. Int J Environ Res Public Health. (2020) 17:124239. 10.3390/ijerph17124239 32545853 PMC7345462

[27] Gutierrez-Robledo LM Avila-Funes JA. How to include the social factor for determining frailty? J Frailty Aging. (2012) 1:13–7. 10.14283/jfa.2012.3 27092932

[28] Tanaka T Takahashi K Hirano H Kikutani T Watanabe Y Ohara Y. Oral frailty as a risk factor for physical frailty and mortality in community-dwelling elderly. J Gerontol A Biol Sci Med Sci. (2018) 73:1661–7. 10.1093/gerona/glx225 29161342

[29] Hironaka S Kugimiya Y Watanabe Y Motokawa K Hirano H Kawai H. Association between oral, social, and physical frailty in community-dwelling older adults. Arch Gerontol Geriatr. (2020) 89:104105. 10.1016/j.archger.2020.104105 32480111

[30] Enderlin C Rooker J Ball S Hippensteel D Alderman J Fisher SJ. Summary of factors contributing to falls in older adults and nursing implications. Geriatr Nurs. (2015) 36:397–406. 10.1016/j.gerinurse.2015.08.006 26343008

[31] Mojtabai R. Insurance loss in the era of the affordable care act: Association with access to health services. Med Care. (2019) 57:567–73. 10.1097/MLR.0000000000001150 31299024

[32] Denninger T. Disability and age-observations from an intersectional perspective. Z Gerontol Geriatr. (2020) 53:211–5. 10.1007/s00391-020-01693-7 32020286

[33] Denison HJ Jameson KA Sayer AA Patel HP Edwards MH Arora T. Poor sleep quality and physical performance in older adults. Sleep Health. (2021) 7:205–11. 10.1016/j.sleh.2020.10.002 33223446

[34] Schafer MH Upenieks L MacNeil A. Disorderly households, self-presentation, and mortality: Evidence from a national study of older adults. Res Aging. (2018) 40:762–90. 10.1177/0164027517741347 29137529

[35] Santiago LM Gobbens RJJ van Assen M Carmo CN Ferreira DB Mattos IE. Predictive validity of the Brazilian version of the Tilburg Frailty Indicator for adverse health outcomes in older adults. Arch Gerontol Geriatr. (2018) 76:114–9. 10.1016/j.archger.2018.02.013 29494871

[36] Pfortmueller CA Lindner G Exadaktylos AK. Reducing fall risk in the elderly: Risk factors and fall prevention, a systematic review. Minerva Med. (2014) 105:275–81. 10.1155/2014/256519 24867188

[37] Kojima G. Frailty as a predictor of future falls among community-dwelling older people: A systematic review and meta-analysis. J Am Med Dir Assoc. (2015) 16:1027–33. 10.1016/j.jamda.2015.06.018 26255098

[38] Gobbens RJ Boersma P Uchmanowicz I Santiago LM. The Tilburg Frailty Indicator (TFI): New evidence for its validity. Clin Interv Aging. (2020) 15:265–74. 10.2147/CIA.S243233 32110005 PMC7041595

