# Supplementary material for: Incidence and predictive value of social frailty among community-dwelling older adults in Southwest China: A prospective cohort study
Source: Front Public Health. 2023 Feb 20;11:1103651. doi: 10.3389/fpubh.2023.1103651 (PMC9986618; doi:10.3389/fpubh.2023.1103651)
Supplement: Supplementary file 1 [file Table_1.pdf]

Supplementary table 1 Longitudinal analyses of SF with Adverse Health events（wave 2）

.

	Variables	Disability				Hospitalization				Fall				Death				PF deterioration			
		Sig.	Exp (B)	95% C.I. Lower	95% C.I. Upper	Sig.	Exp (B)	95% C.I. Lower	95% C.I. Upper	Sig.	Exp (B)	95% C.I. Lower	95% C.I. Upper	Sig.	Exp (B)	95% C.I. Lower	95% C.I. Upper	Sig.	Exp (B)	95% C.I. Lower	95% C.I. Upper
Longitudinal analysis (wave2)	SF	0.142	1.95	0.80	4.74	0.850	1.05	0.63	1.75	0.612	0.78	0.30	2.04	**0.000**	4.89	2.23	10.71	0.442	0.75	0.36	1.56
	Age	**0.001**	1.09	1.04	1.15	-	-	-	-	-	-	-	-	**0.000**	1.12	1.06	1.18	-	-	-	-
	Marital status (having a mate)	-	-	-	-	**0.039**	0.60	0.37	0.97	-	-	-	-	-	-	-	-	-	-	-	-
	Residency Period (year)													0.074							
	3＜x≤10	-	-	-	-	-	-	-	-	-	-	-	-	1	0.82	0.26	2.56	-	-	-	-
	＞10	-	-	-	-	-	-	-	-	-	-	-	-	0.086	2.55	1.03	6.33	-	-	-	-
	Medical Institution Satisfaction	0.050																			
	Normal	0.814	1.48	0.58	3.76	-	-	-	-	-	-	-	-	-	-	-	-	-	-	-	-
	Disappointed	**0.016**	3.52	1.40	8.84	-	-	-	-	-	-	-	-	-	-	-	-	-	-	-	-
	With a confidant	-	-	-	-	-	-	-	-	**0.014**	0.28	0.10	0.77	0.084	7.33	0.77	69.96	-	-	-	-

Abbreviations: SF, Social Frailty;PF, Physical Frailty. We used the stepwise logistic regression model to analyze the relationship of SF with adverse health events. P value was corrected by Benjamini correction method. p-values <0.05 are printed in bold

Supplementary table 2 Longitudinal analyses of SF with Adverse Health events（wave 3）

	Variables	Disability				Hospitalization				Fall				Death				PF deterioration			
		Sig.	Exp (B)	95% C.I. Lower	95% C.I. Upper	Sig.	Exp (B)	95% C.I. Lower	95% C.I. Upper	Sig.	Exp (B)	95% C.I. Lower	95% C.I. Upper	Sig.	Exp (B)	95% C.I. Lower	95% C.I. Upper	Sig.	Exp (B)	95% C.I. Lower	95% C.I. Upper
Longitudinal analysis (wave3)	SF	0.285	0.71	0.37	1.34	**0.041**	0.57	0.33	0.98	0.303	0.74	0.41	1.32	**0.017**	2.22	1.15	4.28	0.903	1.04	0.54	2.01
	Age	-	-	-	-	**0.023**	0.95	0.92	0.99	-	-	-	-	**0.000**	1.15	1.10	1.21	-	-	-	-
	Self-reported memory status													**0.018**							
	Normal	-	-	-	-	-	-	-	-	-	-	-	-	**0.004**	0.28	0.12	0.63	-	-	-	-
	Bad	-	-	-	-	-	-	-	-	-	-	-	-	0.336	0.58	0.27	1.26	-	-	-	-
	With a confidant	-	-	-	-	-	-	-	-	-	-	-	-	**0.043**	14.09	1.09	181.98	-	-	-	-

Abbreviations: SF, Social Frailty;PF, Physical Frailty. We used the stepwise logistic regression model to analyze the relationship of SF with adverse health events. P value was corrected by Benjamini correction method. p-values <0.05 are printed in bold
